# Reaction Center Excitation in Photosystem II: From Multiscale Modeling to Functional Principles

**DOI:** 10.1021/acs.accounts.3c00392

**Published:** 2023-10-16

**Authors:** Abhishek Sirohiwal, Dimitrios A. Pantazis

**Affiliations:** † Department of Biochemistry and Biophysics, Arrhenius Laboratory, Stockholm University, 10691 Stockholm, Sweden; ‡ Max-Planck-Institut für Kohlenforschung, Kaiser-Wilhelm-Platz 1, 45470 Mülheim an der Ruhr, Germany

## Abstract

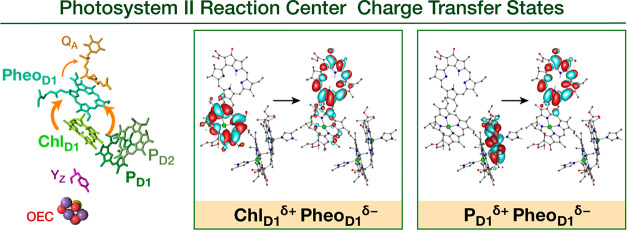

Oxygenic photosynthesis is the fundamental energy-converting process that utilizes sunlight to generate molecular oxygen and the organic compounds that sustain life. Protein–pigment complexes harvest light and transfer excitation energy to specialized pigment assemblies, reaction centers (RC), where electron transfer cascades are initiated. A molecular-level understanding of the primary events is indispensable for elucidating the principles of natural photosynthesis and enabling development of bioinspired technologies. The primary enzyme in oxygenic photosynthesis is Photosystem II (PSII), a membrane-embedded multisubunit complex, that catalyzes the light-driven oxidation of water. The RC of PSII consists of four chlorophyll *a* and two pheophytin *a* pigments symmetrically arranged along two core polypeptides; only one branch participates in electron transfer. Despite decades of research, fundamental questions remain, including the origin of this functional asymmetry, the nature of primary charge-transfer states and the identity of the initial electron donor, the origin of the capability of PSII to enact charge separation with far-red photons, i.e., beyond the “red limit” where individual chlorophylls absorb, and the role of protein conformational dynamics in modulating charge-separation pathways.

In this Account, we highlight developments in quantum-chemistry based excited-state computations for multipigment assemblies and the refinement of protocols for computing protein-induced electrochromic shifts and charge-transfer excitations calibrated with modern local correlation coupled cluster methods. We emphasize the importance of multiscale atomistic quantum-mechanics/molecular-mechanics and large-scale molecular dynamics simulations, which enabled direct and accurate modeling of primary processes in RC excitation at the quantum mechanical level.

Our findings show how differential protein electrostatics enable spectral tuning of RC pigments and generate functional asymmetry in PSII. A chlorophyll pigment on the active branch (Chl_D1_) has the lowest site energy in PSII and is the primary electron donor. The complete absence of low-lying charge-transfer states within the central pair of chlorophylls excludes a long-held assumption about the initial charge separation. Instead, we identify two primary charge separation pathways, both with the same pheophytin acceptor (Pheo_D1_): a fast pathway with Chl_D1_ as the primary electron donor (short-range charge-separation) and a slow pathway with P_D1_P_D2_ as the initial donor (long-range charge separation). The low-energy spectrum is dominated by two states with significant charge-transfer character, Chl_D1_^δ+^Pheo_D1_^δ−^ and P_D1_^δ+^Pheo_D1_^δ−^. The conformational dynamics of PSII allows these charge-transfer states to span wide energy ranges, pushing oxygenic photosynthesis beyond the “red limit”. These results provide a quantum mechanical picture of the primary events in the RC of oxygenic photosynthesis, forming a solid basis for interpreting experimental observations and for extending photosynthesis research in new directions.

## Key References

- SirohiwalA.; NeeseF.; PantazisD. A.Protein Matrix Control of Reaction Center Excitation in Photosystem II. J. Am. Chem. Soc.2020, 142, (42), , 18174–18190.3303445310.1021/jacs.0c08526PMC7582616(1)*The first quantum mechanical description of the lateral and transverse excitonic and charge-transfer states asymmetry in the reaction center of Photosystem II, achieved with multiscale simulations.*
- SirohiwalA.; PantazisD. A.The Electronic Origin of Far-Red-Light-Driven Oxygenic Photosynthesis. Angew. Chem. Int. Ed.2022, 61, e202200356.10.1002/anie.202200356PMC930456335142017(2)*Identification of charge-transfer states implicated in far-red light-driven charge separation, which can be activated in a conformation- and wavelength-dependent manner.*
- SirohiwalA.; NeeseF.; PantazisD. A.How Can We Predict Accurate Electrochromic Shifts for Biochromophores? A Case Study on the Photosynthetic Reaction Center. J. Chem. Theory Comput.2021, 17, (3), , 1858–1873.3356661010.1021/acs.jctc.0c01152PMC8023663(3)*Demonstration of a reference-quality wave function based approach, DLPNO-STEOM-CCSD, for computing excited states of reaction center pigments and for accurately quantifying protein-induced electrochromic shifts in excitation energies.*
- CaponeM.; SirohiwalA.; AschiM.; PantazisD. A.; DaidoneI.Alternative Fast and Slow Charge-Separation Pathways in Photosystem II. Angew. Chem. Int. Ed.2023, 62, e202216276.10.1002/anie.20221627636791234(4)*Elucidation of the kinetics of the two primary charge-separation pathways that operate under different time scales in Photosystem II, using the Perturbed Matrix Method (PMM) approach.*

## 1 Introduction

Photosynthesis is the primary energy conversion process in Earth’s biosphere, harvesting sunlight to create the chemical compounds that sustain life.^5^ Key light-dependent photosynthetic processes take place in an array of transmembrane proteins that work in tandem to generate the proton motive force that drives ATP synthesis. In oxygenic photosynthesis the photosynthetic cascade is initiated at Photosystem II (PSII), a dimeric multisubunit pigment–protein complex that uses light to catalyze water oxidation into molecular oxygen and supply reducing equivalents further along the chain.^6^ A PSII monomer is composed of 20 subunits. The core polypeptides D1 and D2 host the reaction center (RC), the set of six pigments responsible for converting excitation energy into electrochemical potential through charge separation,^7−9^ as well as other redox active cofactors including the all-important oxygen-evolving complex (OEC). Several additional proteins complete the PSII complex to stabilize specific functions and create key water networks required for water oxidation by the OEC.^10^ The excitation energy captured by external light-harvesting complexes is efficiently directed toward the RC via the integral chlorophyll-containing CP43 and CP47 antenna complexes,^9,11^ which are tightly bound around the D1 and D2 chains.

**Figure 1:**
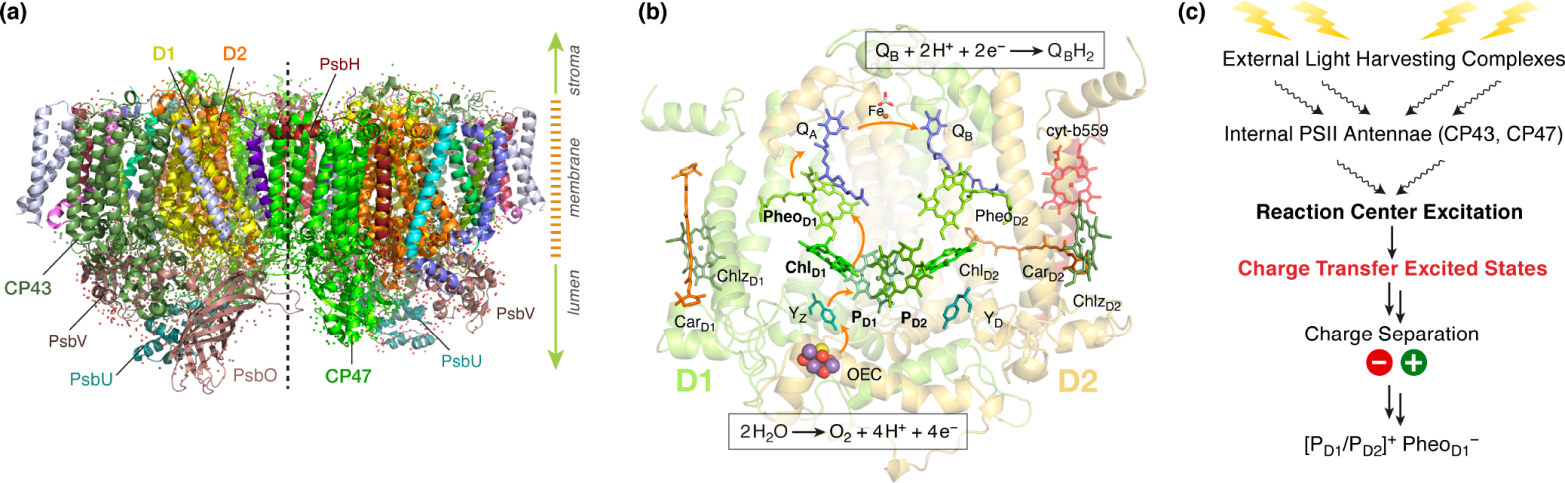
(a) Side view of cyanobacterial Photosystem II dimer with labeling of selected subunits; (b) structure of the reaction center showing important cofactors; arrows indicate the main electron transfer pathway; (c) sequence of major events leading to charge separation in the reaction center of PSII.

The PSII reaction center consists of four Chlorophyll *a* (Chl *a*) and two Pheophytin *a* (Pheo *a*) pigments arranged symmetrically along the D1 and D2 core-polypeptides (Figure 1).^10^ The central pair of Chl *a* molecules (P_D1_ and P_D2_) are flanked by Chl *a* (Chl_D1_ and Chl_D2_) and Pheo *a* pigments (Pheo_D1_ and Pheo_D2_). The D1-side pigments are involved in productive electron transfer, while those on the D2 side are not, possibly playing other roles in photoprotection and regulation.^12^ The D1 and D2 chains contain two additional chlorophylls (Chlz_D1_ and Chlz_D2_, see Figure 1b) in addition to the above-mentioned 6 pigments. Chlz_D1_ presumably participates in excitation energy transfer between CP43 and the RC,^13^ while Chlz_D2_ is suggested to participate in cyclic electron transfer as an intermediate between Cytochrome *b*_559_ and the RC.^14^ Computational studies discussed in the present Account have not considered the Chlz pigments because they are not involved in primary charge separation. Once the RC is excited, electron transfer occurs along D1,^7,15−18^ resulting eventually in the formation of a radical cation species (P_680_^+^) localized in the central P_D1_P_D2_ pair.^12,19^ This cation possesses a redox potential of approximately 1.1–1.3 V, making it the most potent oxidant in biology. It facilitates the oxidation of the OEC and eventually of water (donor side of PSII) through the intermediary redox-active tyrosine D1-Y161 (Y_Z_). On the acceptor side of PSII, electrons are transferred to plastoquinone Q_A_ and eventually to the terminal acceptor plastoquinone Q_B_, leading to formation of the mobile Q_B_H_2_ through a series of proton-coupled electron transfers.^12,20^

Understanding the function of the reaction center requires addressing key questions such as the precise role and properties of the individual pigments, the nature of excited states and particular the nature of charge-transfer (CT) states that initiate charge separation, the origin of functional differences between the D1 and D2 side pigments (functional asymmetry), the localization or delocalization of excitation energy within the reaction center, identification of initial electron donors and acceptors, and the influence of the protein environment on the excited state and redox properties of the reaction center pigments.

Numerous proposals have emerged regarding the identity of the pigments involved in the primary excitation and primary charge separation within the RC. Among them, the multimer model^21^ suggested similar site energies for all RC pigments, promoting delocalization. However, this has difficulty to account for the functional asymmetry of the RC. A major idea involves Chl_D1_ and Pheo_D1_ as the primary electron donor and acceptor, respectively, leading to formation of Chl_D1_^+^Pheo_D1_^–^ as the initial charge-separated state,^1,16,22−25^ before stabilization of the hole on P_D1_ (or P_D1_P_D2_). Chl_D1_ has also been suggested as an intermediary electron acceptor from P_D1_ (formation of a primary P_D1_^+^Chl_D1_^–^ pair),^24,25^ while an initial charge-separated state within the central P_D1_P_D2_ pair has also been considered.^26^ Direct long-range electron transfer from P_D1_P_D2_ to Pheo_D1_ without formation of an anionic Chl_D1_ has also been proposed,^4^ as well as Chl_D1_ and P_D1_ acting in concert as the primary electron donor to Pheo_D1_,^27^ while various studies have raised the possibility of parallel pathways.^2,4,24,25^

Not only the identity of pigments involved in primary charge separation is debated, but also the overall description of the kinetics of charge separation and electron transfer in the RC, where both the nature of kinetic phases and the time constants attributed to specific intermediates are contested. One reason for these difficulties is that experiments are challenged by spectral congestion.^28^ This is partly overcome using protein-pigment complexes extracted from their native assembly. For the PSII RC this involves nonphysiological complexes containing only the D1, D2, and Cyt_b559_ proteins. Such extractions, however, may lead to loss of key spectral features compared to the native assembly,^28^ owing to loss of the native protein fold and potentially of pigments, as exemplified by simulations of the CP47 antenna.^29^

Computational simulations offer indispensable complementary insights into the nature and function of light harvesting complexes.^30,31^ This can even be achieved in a potentially “near-native” state in the case of multiscale simulations of complete protein complexes. However, theoretical approaches require careful consideration of methodological issues to yield reliable insights. This Account discusses important recent progress in this direction, and presents recent findings from our lab on the reaction center of PSII. We focus on the nature of excited states in the PSII RC and describe how a benchmarked multiscale computational approach provided insights into the electronic and structural factors that generate functional asymmetry and enable charge separation.

## 2 Methodologies for Multiscale RC Simulations

In light-harvesting complexes and reaction centers, pigments are diversified by their unique environment within the protein scaffold, such as axial ligation to the central Mg ion, hydrogen bonding, steric effects, and electrostatics. Since intrinsic photophysical properties of bare pigments of the same chemical type are almost identical in the absence of substantial structural distortions, it is the protein environment electrostatics (anisotropic charge distribution) that tunes the spectral properties to enable function, such as directed excitation energy transfer and charge separation.^1,22,30−35^ Therefore, accurate computation of excited state properties of pigments requires explicit consideration of the protein environment and of protein–pigment interactions. Hybrid quantum-mechanics/molecular-mechanics (QM/MM) approaches^36^ allow this to be achieved effectively. In this approach the region of interest, i.e. a pigment and relevant residues, is described at a quantum mechanical level, whereas the rest of the protein is described at the classical (MM) level. Use of a multiscale protocol does not automatically guarantee good results: careful evaluation of methodology at both levels is essential because the results depend sensitively on all components and their interaction.

### 2.1 Atomistic Models of Pigment–Protein Complexes

X-ray crystallography and cryo-electron microscopy (cryo-EM) techniques are commonly used to determine the atomic-level structure of light harvesting complexes. These techniques are typically applied at cryogenic temperatures and rarely inform on conformational dynamics that are crucial for a complete understanding of structure and function. Sample treatment can also lead to nonphysiological changes.^37^ Molecular dynamics (MD) calculations conducted under simulated “physiological” conditions offer a way to obtain advanced understanding of protein dynamics and allow for conformational sampling across a wide range of time and length scales, providing insights into functional states that may not be experimentally accessible. The preparation of MD simulations involves several steps: determining protonation states, embedding the system in a membrane and in a water box, and hydrating internal cavities. Parameterizing noncanonical residues or cofactors for a conventional force field is challenging, particularly for metallocofactors. The computation of electrostatic charges is crucial for parametrization: assigning integer charges based on oxidation states or using restrained electrostatic potential (RESP) computation for redistributing charges on covalently linked ligands are two possible approaches. Experience indicates that the former should be avoided as it leads to long-range Coulombic artifacts.^1^ For PSII, correct modeling of the membrane is also essential for achieving stability over long time scales.^1,38^ After energy minimization, equilibration, and production simulations, snapshots can be extracted from the MD trajectory for QM/MM computations.

### 2.2 Quantum Chemical Methods for Chlorophyll Excited States

**Figure 2:**
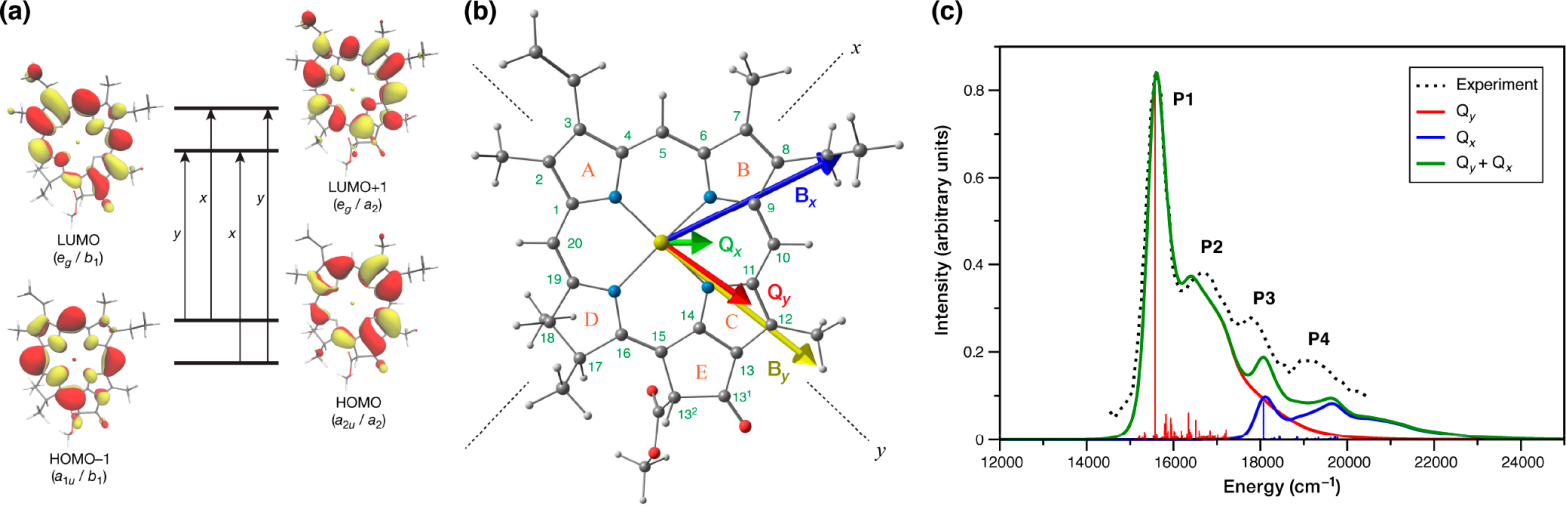
(a) Simplified Chl *a* frontier molecular orbitals associated with excitations in the Q and B bands according to the Gouterman model; (b) orientation of transition dipole moments (DLPNO-STEOM-CCSD) associated with the Q and B transitions of Chl *a*; (c) comparison of the vibronic spectrum of the Q-band of Chl *a* computed using DLPNO-STEOM-CCSD with the experimental gas-phase spectrum.^3^

The low-energy absorption spectrum of chlorins consists of Q and B (Soret) bands.^39^ The Q-band encompasses the two lowest Q_*y*_ and Q_*x*_ excitations (*x* and *y* denote polarization direction in the macrocyclic ring), where Q_*y*_ (also known as site energy) forms the key ingredient for exciton model Hamiltonians. In terms of the Gouterman frontier orbital model (Figure 2),^40^ Q_*y*_ is a π → π* type of excitation (primarily HOMO → LUMO with smaller contribution of HOMO–1 → LUMO+1). Protein matrices influence the energetics of these excitations, leading to spectral tuning, i.e., red or blue shifting relative to the intrinsic site energy. Due to strong conjugation in the macrocyclic ring, even relatively small changes in bond-length alternation due to different description of π-electron localization by quantum chemical methods can have significant impact on orbital energies, which in turn affect site energies and CT states.^39,41^ It has been shown that geometry optimization with different DFT functionals leads to shifts of up to 0.1 and 0.2 eV in the Q and B band, respectively, for the same excited-state method.^39^ Bond-length alteration (BLA) within the macrocycle is a crucial indicator: crystal structures do not accurately depict BLA, therefore quantum mechanical optimization of pigments is essential^41^ for computational studies.

Accurately computing the nature and energetics of Q and B excited states has been a long-standing challenge for computational chemistry. Time-dependent density functional theory (TD-DFT) is commonly used owing to computational efficiency and reasonable accuracy. On the other hand, wave function-based approaches offer higher accuracy but are computationally expensive. Recently, a domain-based local pair natural orbital (DLPNO)^42^ implementation was developed for the similarity transformed equation of motion coupled cluster theory with single and double excitations (STEOM-CCSD).^43^ This enables the application of a highly accurate electron correlation method for the calculation of excited states of large molecules such as chlorophylls. Applying DLPNO-STEOM-CCSD to Chl *a* successfully reproduces the energies and the vibronic features associated with the Q and B bands, as compared to gas-phase experimental data.^39,44^

Comparing density functionals with DLPNO-STEOM-CCSD showed that conventional GGA functionals (such as PBE, BLYP, BP86) fail qualitatively due to inversion of Q_*y*_/Q_*x*_ excitations, while hybrid and range-separated DFT functionals correctly characterized the nature and energetics of Q_*y*_.^3,39^ We stress the necessity of looking beyond the computed energies into the nature of excitations, i.e., by use of natural transition orbitals (NTOs) and the transition dipole moments, to determine their type and thus properly evaluate the quality of results. Range-separated functionals (ωB2PLYP and ωB97X-D3BJ) accurately determine Q_*y*_ energetics. Notably, CC2 and ADC(2), two popular wave function-based approximate approaches, incorrectly mix excitation characteristics for Q_*y*_, with significant contribution of HOMO–1 → LUMO excitations. This is only resolved with spin-component-scaled (SCS) and scaled-opposite-spin (SOS) variants.^45^ In summary, only range-separated DFT functionals and spin-scaled versions of CC2 and ADC(2) correctly predict the nature and energetics of the low-energy spectrum in bare Chl *a*. However, for practical applications in a multiscale framework, it is additionally essential to quantitatively account for protein-induced spectral shifts.

### 2.3 Accurate Calculation of Electrochromic Shifts

**Figure 3:**
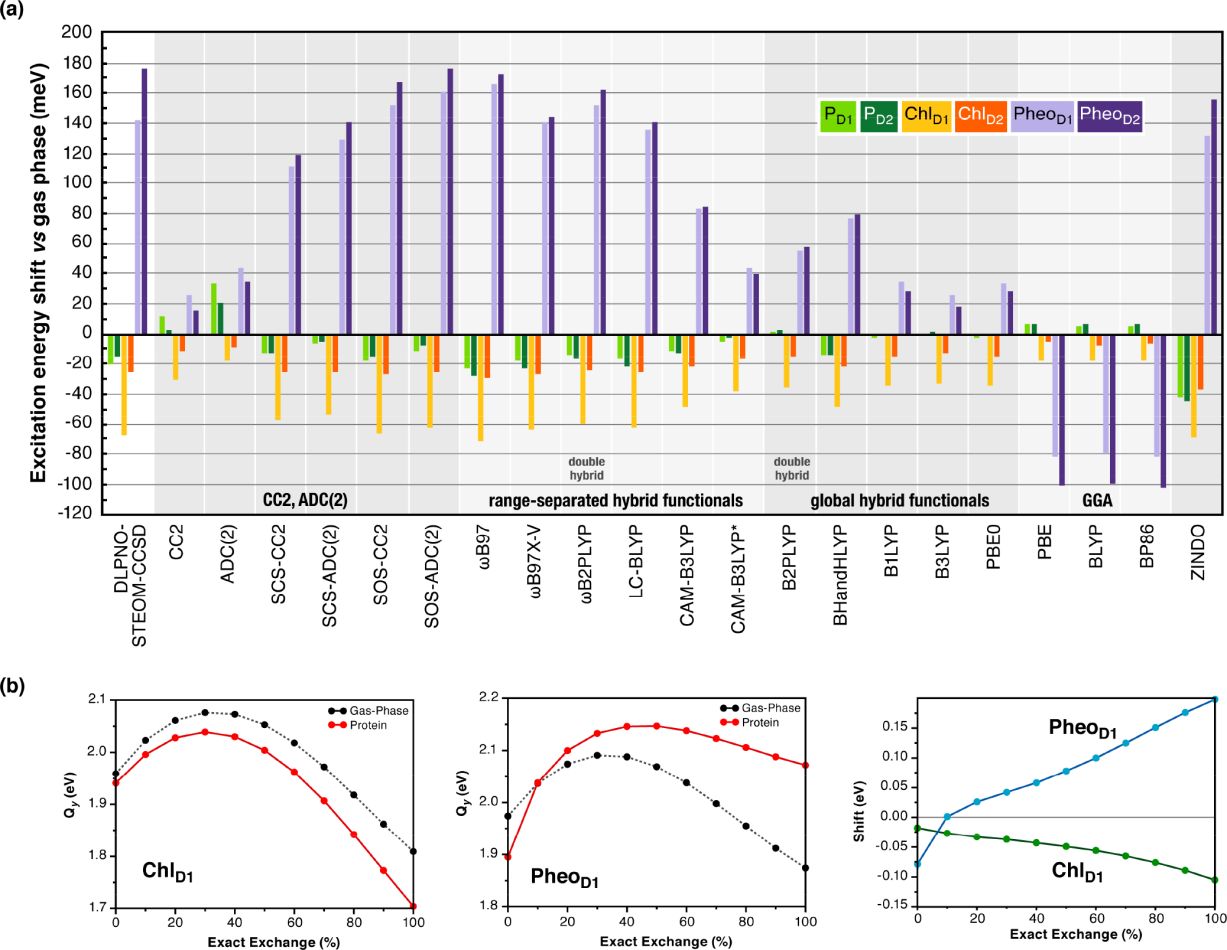
(a) Comparison of electrochromic shifts in S_1_ (Q_*y*_) excitation energies of PSII reaction center pigments obtained different wave function and density functional methods using QM/MM electrostatic embedding; (b) dependence of the first excited state energy and electrochromic shift for the Chl_D1_ and Pheo_D1_ pigments of the PSII RC on the amount of exact (Hartree–Fock) exchange in the B1LYP global hybrid functional.^3^

The protein matrix exerts anisotropic electrostatic influence on pigments, resulting in spectral fine-tuning.^3,46^ We examined various quantum chemical methods to capture electrochromic shifts in the reaction center of PSII, using QM/MM optimization and electrostatic embedding (Figure 3a). DLPNO-STEOM-CCSD is the most reliable wave function method and aligns well with experiment.^3^ According to this, Chl *a* pigments in the RC are red-shifted, while Pheo *a* pigments are blue-shifted. ADC(2) and CC2 vastly underestimate electrochromic shifts and produce incorrect site-energy ordering; the incorrect description of the Q_*y*_ excitation itself may be the reason for this failure. SCS and SOS variants of ADC(2) and CC2 show significant improvement in predicting site-energies and electrochromic shifts, and should be considered as the only acceptable options for practical uses of these methods.

GGA density functionals fail qualitatively, unphysically predicting Pheo_D2_ as the most red-shifted and yielding “ghost” excited states in the low-energy spectrum. Hybrid functionals correctly predict the trend among RC pigments but significantly underestimated the shifts. To investigate the dependence on exact (Hartree–Fock) exchange, we calculated the site energies of Chl_D1_ and Pheo_D1_ pigments with varying exact exchange both in vacuo and in the protein (Figure 3b).^3^ For Chl_D1_, increasing exact exchange up to 30% resulted in a blue-shift in Q_*y*_, while beyond 30%, Q_*y*_ energies are red-shifted. Pheo_D1_ showed more complex behavior, with 0% exact exchange causing a red-shift in protein compared to gas-phase, whereas increase in exact exchange above 10% reverses this trend. The fundamentally distinct behavior for different pigments means it is impossible to select a unique “optimal” exact exchange percentage, and hence global hybrids cannot be used for reliable estimation of electrochromic shifts in pigment–protein complexes. Among range-separated functionals, the popular CAM-B3LYP functional underestimated the electrochromic shifts, particularly for pheophytins, while functionals with 100% long-range exact exchange (ωΒ97, LC-BLYP, ωΒ97X-V, ωB2PLYP) performed the best.

### 2.4 Quantum Chemical Methods for Charge-Transfer States

Understanding charge-transfer (CT) excited states is crucial for studies of light-harvesting and charge separation, including subsequent understanding of electron transfer, quenching, and photoprotection.^47^ Experimental characterization of CT states is hard due to their dark nature, while accurate prediction using quantum mechanics is highly challenging in general, and a weak point of TD-DFT in particular.^48^ This is countered by range-separated functionals that provide improved CT state energetics and potential energy surfaces.^49^ Similar to the observations for electrochromic shifts of individual pigments, comparison with DLPNO-STEOM-CCSD for pigment pairs in the PSII RC shows that range-separated hybrid functionals with 100% long-range exact exchange exhibit correct behavior for CT states in terms of nature, energetics, electrochromic shifts, and (relative) order compared to local excitations. Factoring also the fact that the study of reaction centers requires the simultaneous calculation of multiple pigments to locate all possible CT states, these density functionals appear as the only cost-effective yet still reliable methods that can be currently applied in multiscale studies of photosynthetic reaction centers, at least until much more efficient wave function-based implementations become available.

## 3 Reaction Center Asymmetry

Here we review the results of molecular dynamics and QM/MM calculations on the reaction center of PSII, highlighting the influence of the protein matrix on the excited state energy landscape of individual and oligomeric pigment assemblies and exploring the molecular mechanisms underlying spectral tuning.

### 3.1 Static and Dynamic Structural Asymmetry

**Figure 4:**
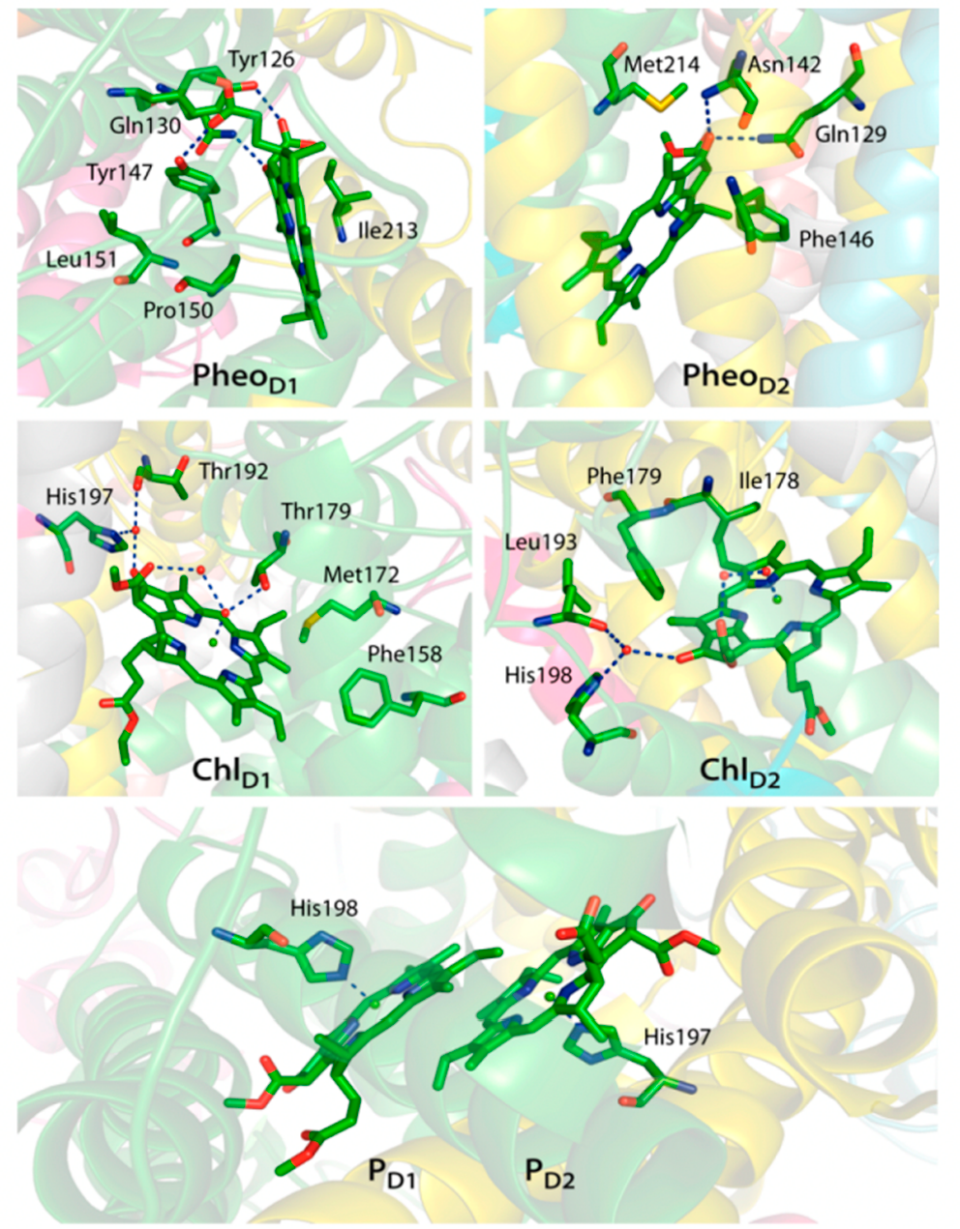
Protein environment around the PSII reaction center pigments. Hydrogen bonding interactions are depicted with dotted lines.^1^

The six pigments (four Chl *a* and two Pheo *a*) of the PSII RC are symmetrically arranged along the core-polypeptides D1 (344 residues) and D2 (342 residues). The chlorophylls in the central P_D1_–P_D2_ pair have weak stacking interactions, with axial ligands from D1 and D2 (D1-His198 and D2-His197, Figure 4), whereas Chl_D1_ and Chl_D2_ have axially ligated water. Structural disparities are further observed in the second coordination spheres: the axial water of Chl_D1_ interacts with D1-Thr179, a neutral-polar residue, whereas Chl_D2_ is associated with the hydrophobic D2-Ile178. Furthermore, the C-13^1^ keto group of both Chl_D1_ and Chl_D2_ forms a hydrogen bond with a water molecule. Regarding pheophytins, the C-13^1^ keto group of Pheo_D1_ interacts with D1-Gln130, while the keto group of Pheo_D2_ can form hydrogen bonds with D2-Gln129 and D2-Asn142. Moreover, C-13^2^-COOCH_3_ and C-17^3^-COOR groups of Pheo_D1_ engage in hydrogen bonds with D1-Tyr147 and D1-Tyr126, respectively, whereas these are replaced by hydrophobic residues (D2-Phe255, D2-Phe125, and D2-Phe146) in Pheo_D2_.

**Figure 5:**
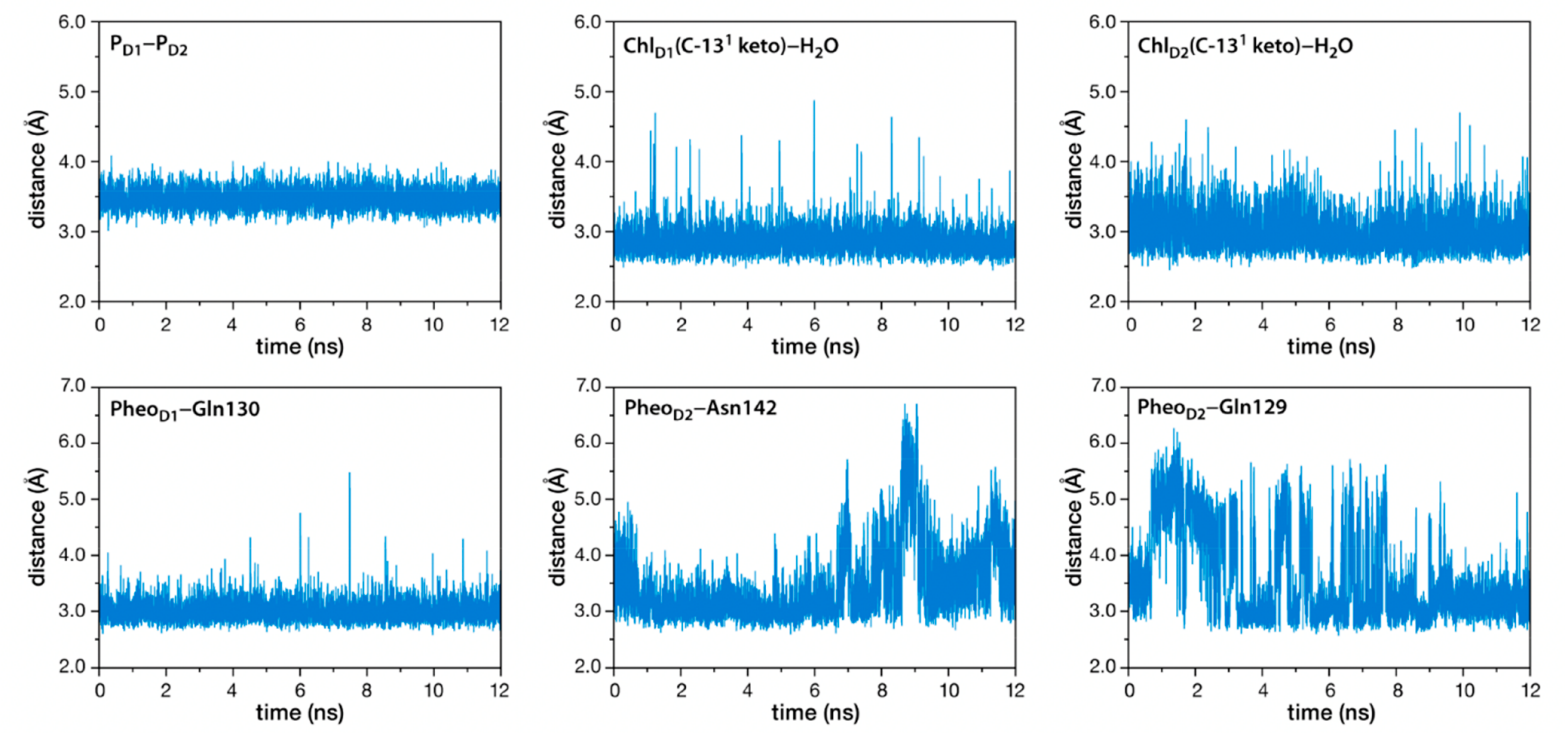
Evolution of selected distances involving reaction center pigments along production MD simulations.^1^

The distinct features of the immediate environment of RC pigments do not serve as predictors of possible differentiation in terms of conformational dynamics. For this we resorted to large-scale MD simulations of the membrane-bound PSII monomer. The simulations show that the D1 and D2 polypeptides do not undergo significant conformational changes.^1,2^ The P_D1_–P_D2_ pair is also very stable. However, intriguing dynamical differences are noticed in pigments along the D1 and D2 chains (Figure 5), for example water bound to the C-13^1^ keto group of Chl_D1_ exhibits tighter binding compared to its Chl_D2_ counterpart. Similar stability variations are observed in Pheophytins, with stronger hydrogen bonding interactions observed in Pheo_D1_ than in Pheo_D2_, specifically with the C-13^1^ keto group. Overall, the protein scaffold enforces less flexibility on the pigments of the active D1 branch compared to the D2 side. The impact of these disparities on RC photochemistry is interesting for further investigation.

### 3.2 Lateral and Transverse Excitation Asymmetry

**Figure 6:**
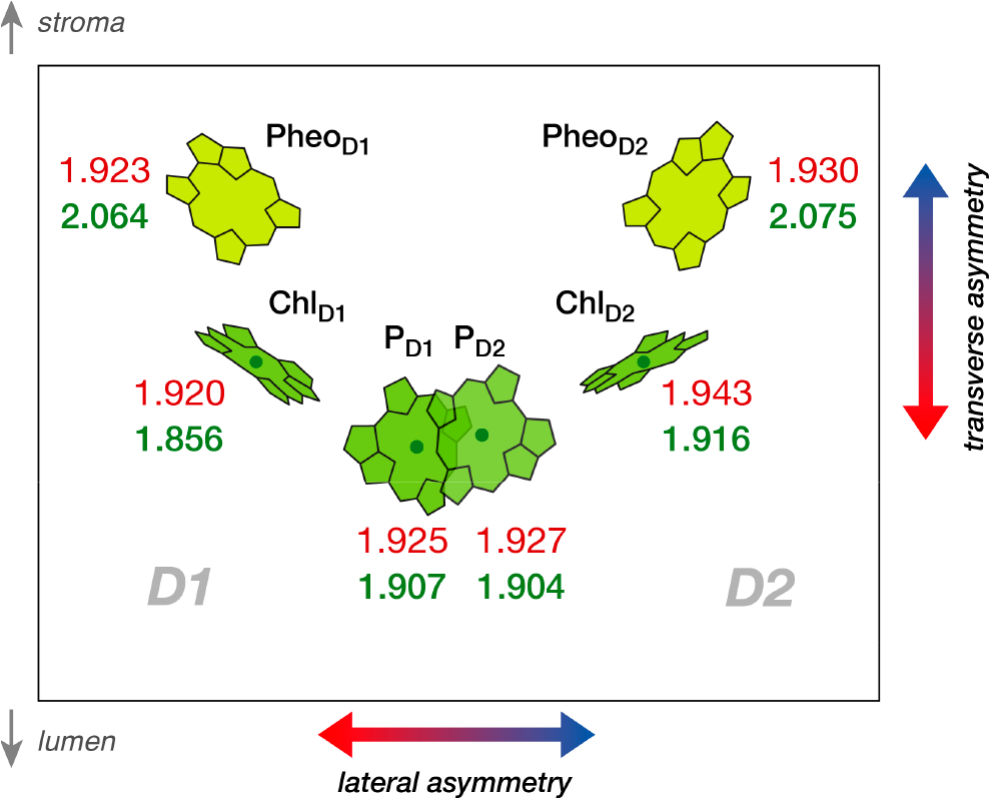
Site energies of the PSII RC pigments *in vacuo* (red) and inside the protein matrix (green) computed at ωΒ97X-D3BJ/def2-TZVP level of theory.^1^ All values in the eV units. The figure also indicates the type of asymmetry induced upon the pigments by protein matrix electrostatics.^1^

The reliable determination of RC pigment site energies is fundamental for understanding excitation energy trapping and charge separation. For this we employed a multiscale QM/MM approach using TD-DFT with the ωΒ97X-D3BJ functional. Initially we investigated the role of the protein in controlling low-energy excited states of individual pigments.^1^ Gas-phase TDDFT calculations on QM/MM optimized geometries (i.e., excluding the protein matrix), reveal similar site-energy values (ranging from 1.920–1.943 eV) for all six pigments (Figure 6). This shows that protein-induced macrocyclic ring strain alone cannot account for RC asymmetry. TD-DFT calculations in the presence of the protein electrostatic environment modify this picture drastically (Figure 6). First, the site energies of all four chlorophylls are red-shifted, while pheophytins exhibit significant blue-shifts. Second, the Chl_D1_ pigment on the D1 side exhibits the most pronounced red-shift and thus the lowest site-energy in the RC.^22,23,50−52^ These results indicate that the transmembrane region of PSII creates two types of electrostatic asymmetry on the RC pigments: transverse (opposite spectral tuning of Chl *a* and Pheo *a* compared to the gas-phase) and lateral asymmetry (differential electrostatic effects along the D1 and D2 sides).

**Figure 7:**
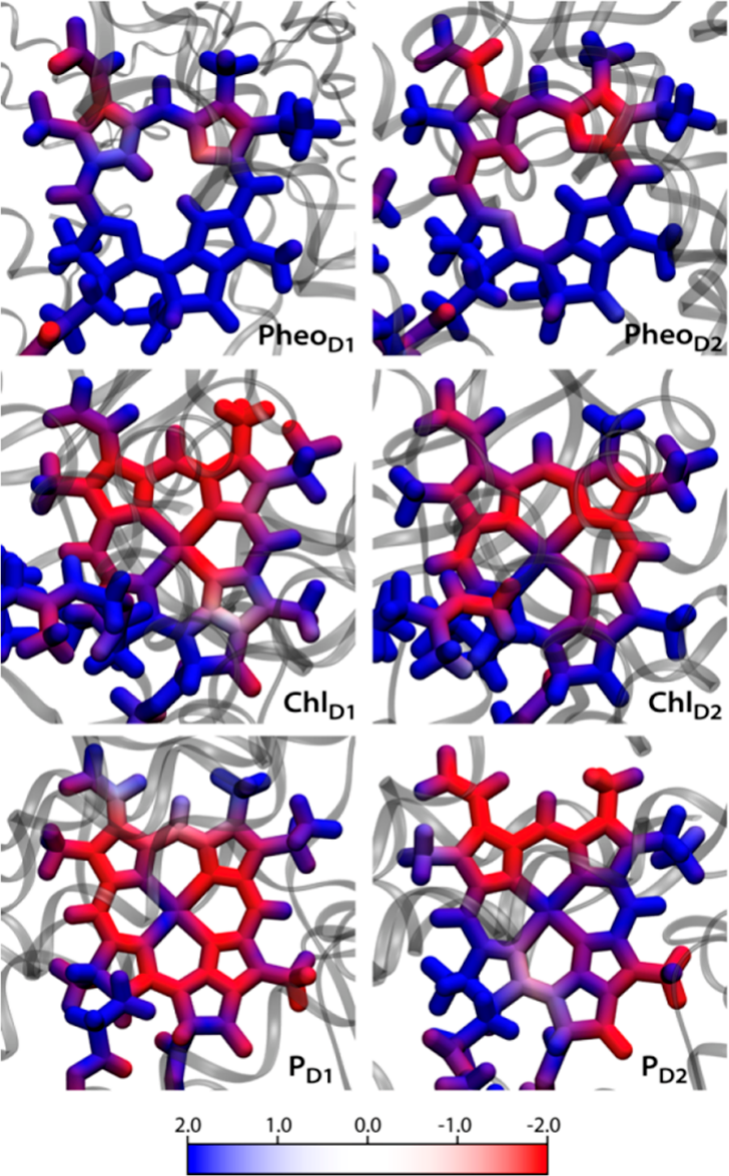
Map of electrostatic potential (in kT/e) projected by the PSII protein matrix on the RC pigments.^1^

These effects can be further analyzed by mapping the protein’s electrostatic potential on the pigments (Figure 7).^46,53^ All Chl *a* pigments are predominantly exposed to negative potential, while the Pheo *a* pigments experience mainly positive potential (transverse asymmetry, i.e., top to bottom, across the membrane). Moreover, the pigments on the D1 and D2 sides have differential electrostatic effects arising from the protein (lateral asymmetry, i.e. left to right, along the membrane). Overall, each RC pigment experiences a unique electrostatic effect, attributed to their specific location and orientation in the transmembrane region. The observed asymmetry in RC pigment site-energies arises exclusively from the electrostatic influence of the protein.

It is possible to trace individual effects on distinct components of the protein matrix. For example, Chl_D1_ experiences a net red shift of approximately 0.064 eV (516 cm^–1^) due to several contributors: D1-Met172 (76 cm^–1^), D1-Phe158 (48 cm^–1^), P_D1_ (79 cm^–1^), and nearby Chloride ions (61 cm^–1^) near the OEC. Conversely, Pheo_D1_ exhibits a net blue-shift of 0.141 eV primarily influenced by D1-Tyr147 (115 cm^–1^), D1-Pro150 (89 cm^–1^), Chl_D1_ (66 cm^–1^), D1-Leu151 (52 cm^–1^), and D2-Ile213 (28 cm^–1^). Interestingly, the red and blue shifting factors differ on the D2 side, indicating the role of localized molecular determinants in lateral asymmetry within the RC. Importantly, while primary factors contributing to these shifts can be identified, the overall changes cannot be fully attributed to a limited set of contributors. Instead, a growing number of residues and cofactors with progressively minor contributions are observed, suggesting global as well as local evolutionary optimization of the electrostatic environment.^22^

## 4 Charge-Transfer States in the Reaction Center

**Figure 8:**
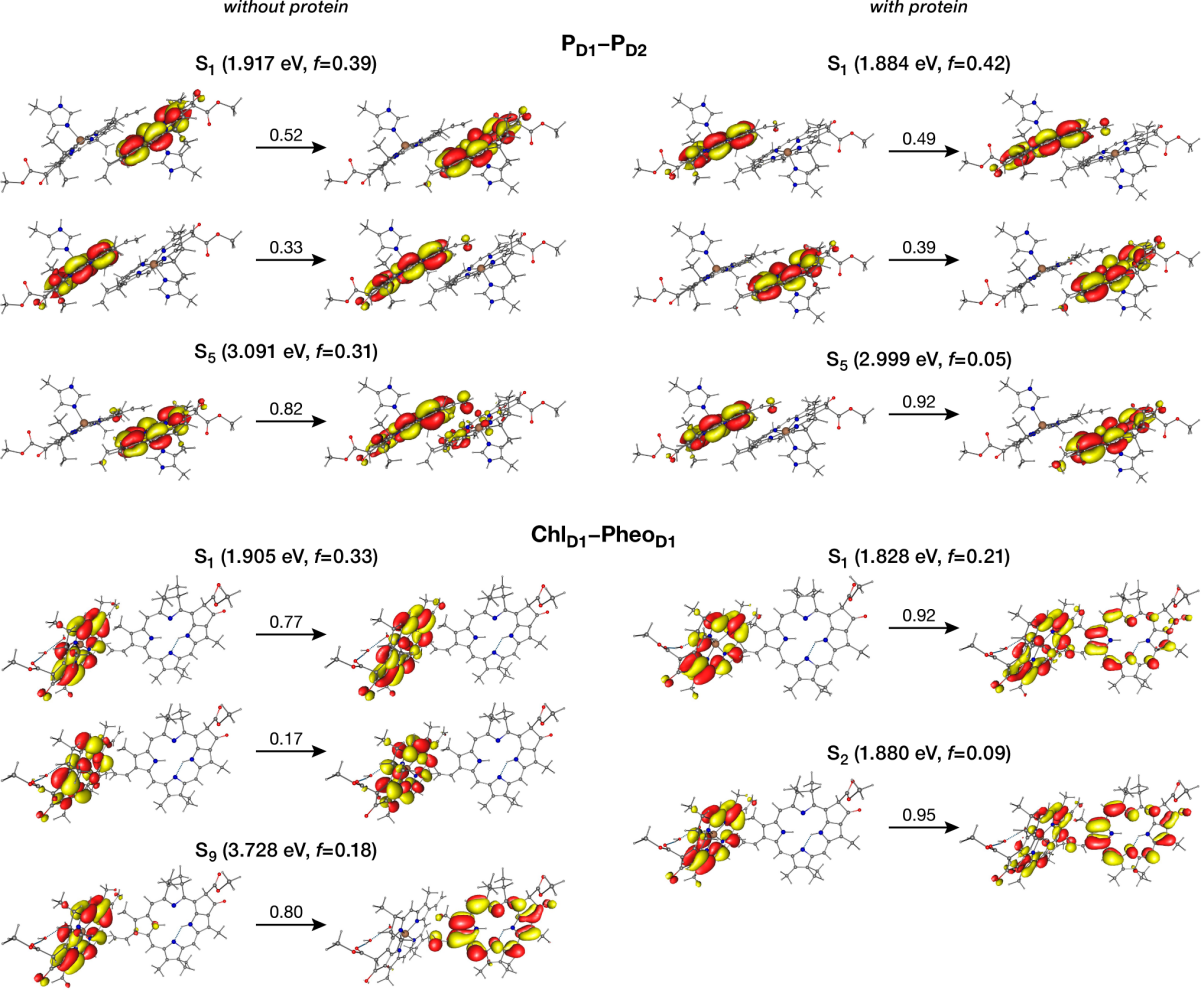
Analysis of the lowest excited state and the first significant CT state in the P_D1_–P_D2_ and Chl_D1_–Pheo_D1_ pairs using Natural Transition Orbitals (NTOs). Vertical excitation energies (in eV), oscillator strengths (*f*) and NTO weights are provided for each state, obtained from ωB97X-D3(BJ)/def-TZVP. The results are compared in absence (*left*) and presence (*right*) of the electrostatic effect of the complete PSII monomer, and show how the protein matrix enables formation of low-lying CT states within the Chl_D1_–Pheo_D1_ pair.^1^

Understanding the nature of local excitations of RC pigments is crucial for insights into excitation energy transfer and trapping, but explicit calculation of CT and mixed CT–exciton states is necessary to understand how primary charge separation is initiated.^54^ In an intermolecular CT state, the electron donor and acceptor are distinct, and excited CT states have a substantial dipole moment, rendering them highly sensitive to protein electrostatics and dynamics. We begin with the central P_D1_–P_D2_ pair, often assumed to be a primary charge-separation site, similar to the bacterial reaction center (BRC).^1^ Initial gas-phase calculations on the QM/MM optimized geometry of the P_D1_–P_D2_ pair show that the lowest energy excited state (S_1_, 1.917 eV) is a combination of local excitation on both P_D1_ and P_D2_, while the first CT state (S_5_) P_D1_^–^P_D2_^+^ is much higher in energy (3.091 eV). When computations are performed in the presence of the protein matrix, the lowest energy excited state red-shifts to 1.884 eV, while maintaining the same nature (Figure 8). The first CT state within the pair is not stabilized in the protein and remains much higher in energy (2.999 eV), but with reversal in the CT direction, P_D1_^+^P_D2_^–^. These results definitively exclude the possibility of charge separation within the P_D1_–P_D2_ pair in PSII.

The Chl_D1_–Pheo_D1_ pair provides a distinct set of results. In gas-phase calculations the lowest energy excited state localized on Chl_D1_ was computed at 1.905 eV, while the first CT state (S_9_, Chl_D1_^+^Pheo_D1_^–^) was at a much higher energy of 3.728 eV. However, when computations are performed within the protein electrostatic field, the picture changes dramatically (Figure 8). The lowest excited state is red-shifted to 1.828 eV with significant mixing of the Chl_D1_^+^Pheo_D1_^–^ CT state. This indicates that the lowest excited state in the RC is not solely localized on Chl_D1_, but is mixed with the Chl_D1_^+^Pheo_D1_^–^ CT state, in line with past suggestions.^26,54,55^ On the D2 side, while a stabilization of the Chl_D2_^+^Pheo_D2_^–^ CT state is observed in the protein versus gas-phase calculations, the lowest excited state remains a local excitation on the Chl_D2_ pigment. Absence of low-lying CT states as well as excitonic asymmetry in past quantum chemical studies of the RC can be ascribed to the neglect of protein matrix electrostatics.^56^ The above results reveal a fundamental exciton–CT asymmetry within the PSII, favoring CT state formation on the D1 side, and identify the low-energy Chl_D1_^+^Pheo_D1_^–^ CT state as the one that initiates charge separation in PSII.^1^

To further map the low-energy spectrum of the active branch, we employed a single contiguous QM region comprising the P_D1_–P_D2_–Chl_D1_–Pheo_D1_ tetramer.^2^ TD-DFT calculations were performed for 22 snapshots obtained from a 200 ns classical MD production simulation. Our findings reveal two types of low-energy state: (a) local excitations (LE) on Chl_D1_ and P_D1_P_D2_, and (b) Chl_D1_^+^Pheo_D1_^–^ and P_D1_^+^Pheo_D1_^–^ CT states. The LE on Chl_D1_ exhibits slight mixing with the Chl_D1_^+^Pheo_D1_^–^ CT state. The energetics of CT states are significantly influenced by protein dynamics. The LE on Chl_D1_ spans the red region (1.772–1.839 eV), whereas the Chl_D1_^+^Pheo_D1_^–^ CT state samples both red and far-red regions (1.474–1.893 eV). The Chl_D1_^+^Pheo_D1_^–^ state exhibits notable oscillator strength due to its mixing with the LE of Chl_D1_. Conversely, the P_D1_^+^Pheo_D1_^–^ state has reduced oscillator strength (dark state) due to minimal overlap between donor and acceptor orbitals. It is also much more sensitive to protein dynamics, sampling a wide energy range of 1.380–2.305 eV.^2^

It is noted that far-red light (700–800 nm) was conventionally considered outside the range utilized by PSII, but photosynthetic activity has been reported in the far-red region without changes in pigment composition,^57−60^ and a cooperative role of far-red light in photosynthetic activity has been demonstrated.^61^ The computational identification of the two CT states that can sample the far-red region assisted by protein conformational dynamics (i.e., lower than the site energy of the red-most Chl_D1_) offers a view into a molecular mechanism for direct far-red excitation and charge separation within the PSII RC, but further investigation is needed to understand the cooperativity of red and far-red light.

Overall, the findings described above show the presence of precisely two types of low-lying CT states in the PSII RC, Chl_D1_^+^Pheo_D1_^–^ and P_D1_^+^Pheo_D1_^–^. Chl_D1_ is identified as the most likely primary donor and Pheo_D1_ as the exclusive acceptor. But the results are equally important for what they exclude, which is the possibility of energetically accessible CT states within the P_D1_–P_D2_ pair and of CT states that involve Chl_D1_ as intermediary electron acceptor.^1,2^

## 5 Thermodynamics and Kinetics of Primary Charge-Separation Pathways

The computational studies discussed above identified two CT states of potential physiological significance in the active D1 branch of PSII, with Chl_D1_ and P_D1_ as two possible primary electron donors to Pheo_D1_. To further investigate the nature and kinetics of charge separation processes we used multiscale Perturbed Matrix Method (PMM) simulations, which explicitly account for protein dynamics and flexibility.^4^ In the PMM approach the region of interest is considered at a reference quantum level whereas the environmental effects are included as an electrostatic perturbation to the gas-phase properties of the quantum center, providing the ability to use information from long MD trajectories without having to recompute properties at the QM level for every snapshot.^62^

**Figure 9:**
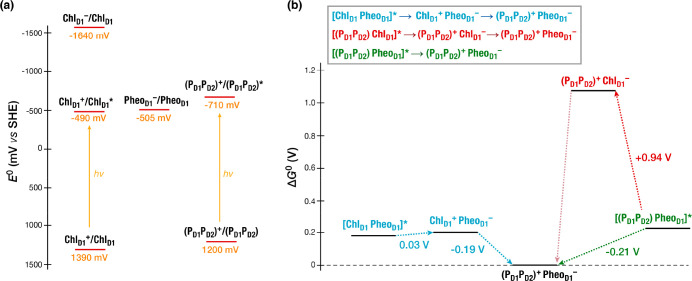
(a) Standard reduction potentials (*E*^0^ vs SHE) of pigments in the D1-branch of the PSII RC in their ground and excited states, calculated using the multiscale MD-PMM approach. Yellow lines represent free energy change upon light absorption. (b) Gibbs free energy changes (Δ*G*^0^) associated with different charge separation pathways.^4^

Computed standard reduction potentials (*E*^0^) of the RC components were 1200 ± 35 mV for the P_D1_P_D2_ pair and 1390 ± 35 mV for Chl_D1_^+^/Chl_D1_ (Figure 9). The *E*^0^ values for the central pair align with the experimentally determined range of 1100–1300 mV.^63^ Our results indicate that the hole is more stable on P_D1_P_D2_ than Chl_D1_. In terms of standard free-energy changes (Δ*G*^0^) for charge-separation scenarios involving Chl_D1_ and P_D1_P_D2_ as initial electron donors, in the Chl_D1_ pathway electron transfer from Chl_D1_ to Pheo_D1_ leads to the Chl_D1_^+^Pheo_D1_^–^ charge-separated state with Δ*G*^0^ close to zero (+0.03 ± 0.07 V), followed by exothermic electron transfer from P_D1_P_D2_ to Chl_D1_^+^ (Δ*G*^0^ = −0.19 ± 0.07 V), resulting in the [P_D1_P_D2_]^+^Pheo_D1_^–^ state. In the P_D1_P_D2_ pathway, long-range electron transfer from P_D1_P_D2_ to Pheo_D1_ is highly favorable (Δ*G*^0^ = −0.21 ± 0.07 V), without involving anionic Chl_D1_ as intermediate (Δ*G*^0^ = +0.94 ± 0.07 V). It is thus reaffirmed that both Chl_D1_ and P_D1_P_D2_ can serve thermodynamically as primary electron donors to the primary acceptor Pheo_D1_. However, to understand the competition between the two pathways it is essential to assess the corresponding kinetics.

Electron transfer kinetics were computed using the PMM approach. Both pathways exhibit biexponential behavior. The Chl_D1_ pathway has time constants of τ_1_ = 163 ± 50 fs (60%, fast) and τ_2_ = 2.0 ± 0.4 ps (40%, slow), while the P_D1_P_D2_ pathway τ_1_ = 185 ± 20 ps (62%, fast) and τ_2_ = 4.2 ± 0.8 ns (38%, slow). The fast Chl_D1_ pathway matches subpicosecond and 1–5 ps experimentally reported components, while the slow, long-range pathway involving the central pair as donor aligns with reported slow components (100–350 ps and a few nanoseconds).^16,17,24^

**Figure 10:**
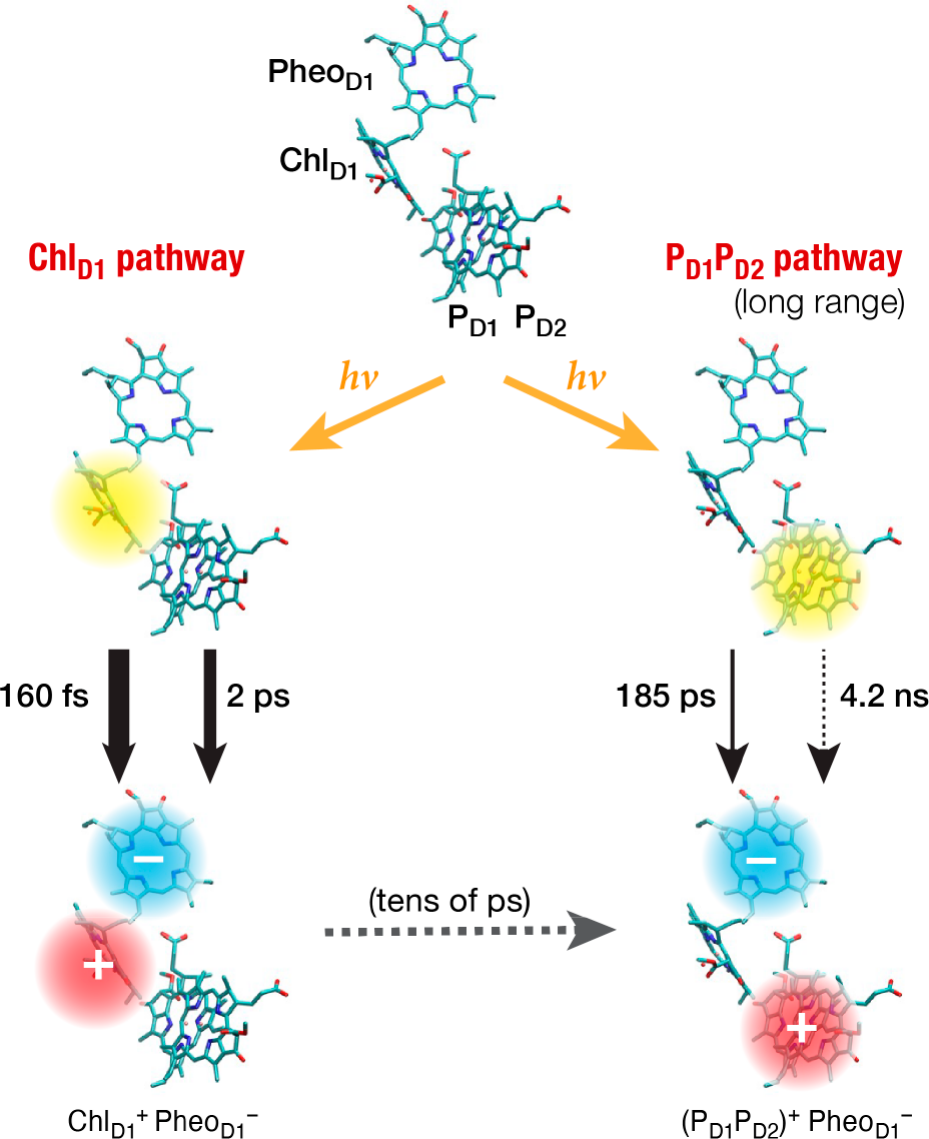
Scheme depicting primary localization of exciton (yellow areas) and computed time constants for the primary electron transfer events in the two thermodynamically viable charge separation pathways. The sites of primary charge-separation are depicted with red (positive) and blue (negative) areas. Both pathways resolve to (P_D1_P_D2_)^+^Pheo_D1_^–^.^4^

Although the computational picture will require further refinement with consideration of the complete kinetic profile, including exciton relaxation and back-energy transfer, our thermodynamics and kinetics analysis confirms the existence–in principle– of two primary charge separation pathways in the RC of PSII, but with distinct time scales (Figure 10). The Chl_D1_^+^Pheo_D1_^–^ pair is clearly favored as the primary charge separated state and the corresponding pathway vastly outcompetes kinetically the long-range ET alternative. Our simulations so far do not allow us to hypothesize about the factors and conditions that might render the slow pathway significant enough to be considered as an alternative pathway of physiological significance.

## 6 Conclusions and Perspectives

Understanding the function of photosynthetic reaction centers at the electronic-structure level is a quest situated at the frontiers of molecular science, at the intersection of grand-challenge theoretical chemistry and biology. In this Account, we presented a brief overview of recent achievements in the area of computational modeling of the electronic structure and photochemistry of the reaction center of Photosystem II using multiscale quantum/classical methods. Essential components of the computational protocol include the explicit consideration of long-range electrostatics, which uniquely determine the properties of the protein-embedded pigments, and the use of properly benchmarked quantum chemical methods (in this case against the DLPNO-STEOM-CCSD method that emerges as a new standard in the field) in order to correctly predict the electrochromic shifts induced by the protein matrix as well as the nature and energetics of pigment-specific properties and of charge-transfer states. Building on the newly developed computational protocol, we have been able for the first time to provide a successful, experimentally consistent, electronic structure description of the primary events in the reaction center of oxygenic photosynthesis, together with the theoretical tools necessary to achieve this description.

The insights obtained so far indicate the presence of two types of excitonic asymmetry at work within the PSII RC (lateral and transverse), and assign its origin exclusively to protein matrix electrostatics. Our calculations identify precisely two possible productive charge separation pathways, fixing the identity of primary donor and acceptor pigments. Using molecular dynamics coupled with the PMM approach further enabled us to characterize the kinetics of the two primary charge separation pathways. The results not only allow us to rationalize a long series of experimental observations–among others, offering a molecular mechanism for far-red-light-driven charge separation–but also to confidently reject a large number of alternative hypothetical scenarios regarding the primary events in the PSII RC discussed in the field during the last 20 years. The results not only make connections to a vast number of experimental studies that cannot be fully reviewed here^1,17,23−25,50,64−67^ but also relate directly to very recent important spectroscopic investigations of the PSII RC.^27,68^ Now that fundamental methodological principles have been clarified, we anticipate that the approach that worked so well for the PSII RC will have implications for computational research activity in multiple relevant directions, including other photosystems and light-harvesting complexes, variants and mutants, but also the burgeoning field of artificial photosynthesis and beyond.
